# Clinical characteristics, factors associated with urinary tract infection and outcome in acutely admitted patients with infection; an exploratory cross-sectional cohort study

**DOI:** 10.1016/j.heliyon.2024.e32815

**Published:** 2024-06-11

**Authors:** Mathias Amdi Hertz, Helene Skjøt-Arkil, Anne Heltborg, Morten Hjarnø Lorentzen, Mariana Bichuette Cartuliares, Flemming S. Rosenvinge, Stig Lønberg Nielsen, Christian Backer Mogensen, Isik Somuncu Johansen

**Affiliations:** a Department of Infectious Diseases, Odense University Hospital, University of Southern Denmark, Denmark; b Research Unit of Infectious Diseases, Department of Clinical Research, University of Southern Denmark, Denmark; c Department of Regional Health Research–Odense, University of Southern Denmark, Denmark; d Emergency Department–Aabenraa, University Hospital of Southern Denmark, Denmark; e Department of Clinical Microbiology, Odense University Hospital, University of Southern Denmark, Denmark; f Research Unit of Clinical Microbiology, University of Southern Denmark, Denmark

**Keywords:** Urinary tract infection, Clinical characteristics, Symptoms, Urine dipstick analysis, Length of stay, Mortality

## Abstract

**Background:**

Urinary tract infections (UTIs) remain a leading infectious disease cause of admission to the emergency department (ED) and antibiotic prescription. Heterogeneity of disease presentation challenges early diagnostics, leading to improper antibiotic prescription and delayed diagnosis. Prior studies have relied on positive urine cultures for diagnosis, but its performance suffers from false positives and false negatives. This study aimed to identify factors associated with UTIs and describe patient characteristics and outcomes while not using positive urine culture as an obligatory part of diagnosis.

**Methods:**

Adult patients admitted to the ED suspected of infection were prospectively included in an exploratory cross-sectional cohort study. An expert panel retrospectively determined the final diagnosis. Factors associated with a UTI were identified using univariate and multivariate logistic regression analysis, outcomes were evaluated with adjusted Cox regression analysis, and length of stay was compared with a zero-inflated negative binomial logistic regression model.

**Results:**

Of 966 patients who were enrolled, 200 were diagnosed with a UTI by the expert panel. We found a significant association between a UTI diagnosis and the typical UTI symptoms: dysuria (OR 7.8), change of urine appearance (OR 3.9), suprapubic or flank pain (OR 3.7), and increased urinary frequency (OR 3.2). Urinary dipstick analysis for white blood cells (WBCs) (OR 6.0–24.0), nitrite (OR 4.7), and blood (OR 3.6–12.0) was also significantly associated. Subgroup analysis of urinary dipstick analysis of men and women still showed significance in both groups. No significant difference in outcome or length of stay was found.

**Conclusion:**

Typical UTI symptoms are associated with a UTI diagnosis, which underlines the importance of exploring a patient's medical history. Urinary dipstick analysis for WBC, nitrite, and blood is also strongly associated and should be considered when evaluating patients admitted to the ED with suspicion of infection.

## 1 Introduction

Urinary tract infections (UTIs) continue to be a leading infectious disease cause of admission to the emergency department (ED) and antibiotic prescription [1,2]. However, the current diagnostic tools suffer from poor performance [3,4]. Better diagnostic tools will improve diagnostic precision, leading to more appropriate empiric antibiotic prescriptions and reducing unnecessary examinations [5,6].

Every year, UTIs cause more than 2 million ED visits in the United States and cause significant mortality and morbidity, especially in vulnerable populations [7,8]. Globally, an estimated 150 million UTIs occur annually, with a lifetime prevalence of 50 % among women and 5 % among men [9]. In Denmark, over 15,000 patients are admitted due to UTIs every year, with an average length of stay of 2.9 days in 2018 [10].

Antibiotic prescription for UTIs worldwide has contributed to an increase in antimicrobial resistance [7,11]. This rise is partly driven by inappropriate antibiotic prescription, leading to increased rates of antimicrobial resistance, an increased risk of subsequent UTI, risk of *Clostridioides difficile* infection, and pharmacological side effects [[12], [13], [14]]. Better diagnostic capabilities have been shown to improve antibiotic prescription in the ED, reducing antimicrobial resistance [15].

Urinary dipstick analysis (UDA) is used to detect both inflammation and the presence of bacteriuria but is an imperfect test with varying accuracy between studies and is susceptible to sampling bias [16,17]. Most studies on the accuracy of UDA have been done against urine culture as the gold standard or as an obligatory part of the criteria for having a UTI. Additionally, recent studies have shown that UDA's positive and negative predictive values differ according to sex, complicating its use [18].

Many diagnostic scoring systems and algorithms have been proposed, combining genitourinary symptoms and urine analysis, but they have yet to achieve general acceptance [[19], [20], [21]]. This low acceptance is likely due to the broad spectrum of disease presentation and severity of UTI, from acute cystitis with dysuria but no systemic symptoms to severe septicemia requiring intensive care [22]. Most studies, therefore, use a rigid definition of UTIs where a positive urine culture is obligatory for a UTI diagnosis. However, high rates of asymptomatic bacteriuria in elderly women and negative urine cultures in high-risk symptomatic patients indicate that urine culture is not a perfect test [1].

### 1.1 Objectives

Since prior studies described patients with UTIs generally defined as having positive urine culture and genitourinary symptoms, we wanted to thoroughly describe UTI patients where the definition reflected the clinical reality.

The objective of the study was to identify factors (symptoms, comorbidities, and clinical findings) associated with UTI based on an expert panel review with no requirement of positive urine culture and to describe patient characteristics, clinical findings, and patient outcomes.

## 2 Methods

### 2.1 Study design

We performed an exploratory cross-sectional study with prospective data collection to identify factors associated with being diagnosed by an expert panel with a UTI in EDs. In addition, we did a cohort study to describe outcomes: length of stay, mortality, intensive care unit admission, and readmission of patients admitted with a UTI.

This study is a sub-study of the Improved Diagnostics of Infectious Diseases in Emergency Departments (INDEED) trial [23].

The study was designed and reported in accordance with the STROBE guidelines [24].

### 2.2 Setting

Patients were recruited from medical EDs at three hospitals in the Region of Southern Denmark: Hospital Sønderjylland in Aabenraa and Sønderborg, Hospital Lillebælt in Kolding, and Odense University Hospital in Odense on weekdays from 8 a.m. to 8 p.m. from March 1, 2021 to February 28, 2022. The three hospitals covered urban and rural areas with a population of approximately 775,000. All participating hospitals are part of the Danish tax-paid universal healthcare system and receive all patients from a well-defined catchment area.

Six project assistants with healthcare education monitored new patients admitted to the ED via the patient management system Cetrea Anywherium (Getinge Cetrea). The project assistants included the patient if an infection was suspected after the treating physician's initial assessment. The suspected focus, pulmonary, urinary tract, or unknown or other, was registered.

When multiple patients were eligible at the same time, but time restraints did not allow for the inclusion of them all, patients were screened and offered participation in the order in which the treating physician assessed them to avoid selection bias.

### 2.3 Variables and data sources

After consent, the project assistant used a structured data collection tool to interview patients and collected data on sex assigned at birth, current symptoms and duration, smoking, and history of previous UTI, pneumonia, or erysipelas.

Data compiled from the medical records were: Vital signs (respiratory rate, oxygen saturation, supplemental oxygen, heart rate, blood pressure, temperature, and Glasgow Coma Scale, clinician's findings (lung auscultation and abdominal examination), prior infections, comorbidities including previous or current cancer, and results of the initial laboratory results (hemoglobin, white blood cells (WBCs), platelets, neutrophilocytes, lymphocytes, albumin, creatinine, blood urea nitrogen, sodium, bilirubin, and c-reactive protein). A medical laboratory assistant took blood tests as part of the normal admission to the ED. The blood test essays and cut-offs were similar across all three hospitals. UDA was performed by the project assistant, except for a few analyses done by the nurse caring for the patient. Siemens Multistix 7 was used for all dipstick tests and automatically read by Siemens Clinitek status + analyzer. Only a few cases were manually read.

A follow-up assessment at 30 days was made to register if the patient had been discharged from the hospital and the duration of the hospital stay. Finally, a follow-up assessment at 90 days recorded mortality, readmissions, and intensive care unit admission and duration.

From all the variables collected in the INDEED trial, we selected the above based on prior literature and clinical experience. We chose only variables available in the ED upon admittance and used a low threshold for including variables because of the exploratory study design.

Project assistants registered all variables in real-time in an online data collection tool (REDCap version 10.8.3 to version 12.2.1 by Vanderbilt University, TN, US).

An expert panel consisting of pairs of infectious diseases and emergency medicine specialists on each site was established. The expert panel pairs reviewed all medical records and determined the ultimate diagnosis based on information within seven days after the inclusion of the patient. A consensus was reached in case of conflicting diagnosis. No algorithm or scoring system was used to define UTIs. The diagnosis was solely based on the expert panel's evaluation of which infection was most likely in each case. Thus, a diagnosis of UTI was possible even with a negative urine culture. The diagnosis was unavailable for the project assistants during data collection.

### 2.4 Statistical methods

Descriptive statistics (proportion for categorical and median (IQR) for continuous variables) of patients’ characteristics were reported.

Univariate logistic regression analysis of every selected variable was performed. The linearity assumption of continuous predictor variables was tested using Box-Tidwell analyses. P-values <0.05 were considered to compromise the linearity assumption, and the variable was categorized for further analysis. Secondly, logistic regression models adjusting for age and sex were implemented. We checked for correlation between the significant variables. We excluded the clinically least used one of each pair for the multiple logistic regression model if they had a correlation factor over 0.5.

Variables demonstrating statistical significance (p < 0.05) in the univariate logistic regression analyses were analyzed in a multiple logistic regression model. The analyses were rerun to examine the effect of outliers, excluding absolute values of Pearson's standardized residual larger than 2.

As a result of the study design, every patient with a final diagnosis of “not infected” would end up in the non-UTI group. These non-infected patients could introduce false associations with UTIs for variables associated with infections. To avoid this, we repeated the fully adjusted analysis but excluded patients without infection and compared the results.

Previous studies have demonstrated that UDA results vary between sexes; hence, the analyses were repeated and stratified by sex.

Cox regression was used to estimate the hazard rate of mortality, readmission, and intensive care admission. We adjusted for age and sex. The follow-up time for intensive care admission was from the date of ED contact until 30 days after and 90 days for readmission and death.

Length of stay was compared with a zero-inflated negative binomial logistic regression model to account for an excess number of hospital contacts with a zero day length of stay. All statistical analyses were carried out using (StataCorp. 2021. *Stata Statistical Software: Release 17*. College Station, TX, US: StataCorp LLC.)

## 3 Results

### 3.1 Participants

**Fig. 1:**
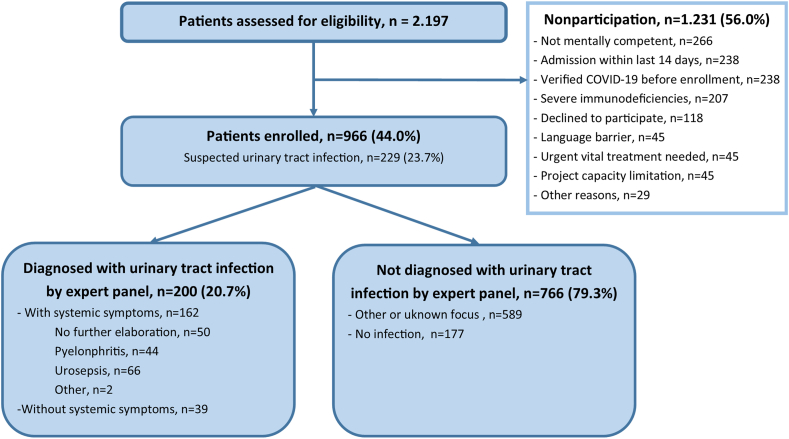
Flow chart with patient profiles.

We screened 2197 patients for inclusion. Of these, 1231 patients either declined or met one or more exclusion criteria. The remaining 966 patients were enrolled in the study, of which 200 (20.7 %) were diagnosed with a UTI by the expert panel. No patients were lost to follow-up (Fig. 1).

#### 3.1.1 Descriptive data

Table 1 shows the characteristics of the study population. The median age for patients with UTI was 76 years (IQR 17), with a slight predominance of men (58 %). The most commonly reported symptoms were fever (73.0 %), increased urinary frequency (36.4 %), change in urine appearance (31.0 %), and dysuria (30.0 %). These symptoms were more prevalent in the patients diagnosed with a UTI than in the non-UTI group. Previous UTI was reported in 66.9 %, and 23.5 % had a permanent urinary catheter. Clinical examination found suprapubic or flank tenderness in 21.0 %, while 59.5 % had a temperature of 37.5 °C or above. UDA was positive for blood in 88.9 %, WBC in 84.7 %, and nitrite in 32.3 %. The median C-reactive protein was 137.5 mmol/L (IQR 130.5), and the median WBC was 12.7 10E9/L (IQR 7.3).

**Table 1:** Characteristics of patients admitted to emergency department with suspicion of infection stratified by urinary tract infection.

**Patient characteristics**		Urinary tract infection	No urinary tract infection
n = 966 unless stated otherwise.		(n = 200)	(n = 766)
		No. (%)	No. (%)
Age, median (IQR), years		76 (17)	72 (23)
Sex	Male	116 (58 %)	405 (52.9 %)
Comorbidities and Previous diseases			
Previous Urinary tract infection, n = 863		123 (66.8 %)	346 (49.8 %)
Chronic kidney disease		21 (10.5 %)	39 (5.1 %)
Kidney stones or Previous kidney stones		14 (7.0 %)	22 (2.9 %)
Urine incontinence		10 (5.0 %)	17 (2.2 %)
Urine retention or prostate hypertrophy		29 (14.5 %)	51 (6.7 %)
Indwelling urine catheter		47 (23.5 %)	39 (5.1 %)
Other urinary tract comorbidities		15 (7.5 %)	33 (4.3 %)
Previous or current cancer of the urinary tract		30 (15 %)	42 (5.5 %)
Reported symptoms			
Sensation of fever		146 (73.0 %)	475 (62.0 %)
Suprapubic or flank pain		35 (17.5 %)	55 (7.2 %)
Increased urinary frequency, n = 922		68 (36.4 %)	115 (15.7 %)
Dysuria, n = 922		56 (30.0 %)	39 (5.3 %)
New urine retention, n = 922		41 (21.9 %)	32 (4.4 %)
New urine incontinence, n = 922		42 (22.5 %)	49 (6.7 %)
Change of urine appearance, n = 922		58 (31.0 %)	77 (10.5 %)
Change of urine smell, n = 922		40 (21.4 %)	41 (5.6 %)
Confusion, n = 927		56 (29.6 %)	156 (21.1 %)
Any of the above symptoms, n = 915		160 (86.5 %)	508 (69.6 %)
Initial vital signs at emergency department			
Respiratory rate, median (IQR), n = 960		18 (4)	19.5 (6)
Periferal oxygen saturation, median (IQR), %, n = 962		97 (3)	96 (4)
Heart rate, median (IQR) n = 965		89 (27)	89 (24)
Mean arterial blood pressure, mean (SD), mmHg, n = 963		90.8 (16.3)	94.9 (15.4)
Temperature, mean (SD), °C, n = 961		37.8 (1.0)	37.4 (0.95)
Initial clinical findings			
Palpatory flank or suprapubic tenderness, n = 966		42 (21.0 %)	49 (6.4 %)
Initial urine dipstick results			
Urine white blood cells, n = 812	Negative	29 (15.3 %)	418 (67.1 %)
	+	32 (16.9 %)	81 (13.0 %)
	++	62 (32.8 %)	75 (12.0 %)
	+++	30 (15.9 %)	29 (4.7 %)
	++++	36 (19.0 %)	20 (3.2 %)
Urine nitrite, n = 812	Positive	61 (32.3 %)	56 (9.0 %)
Urine blood, n = 811	Negative	21 (11.1 %)	283 (45.5 %)
	+/− or +	53 (28.0 %)	188 (30.2 %)
	++	45 (23.8 %)	82 (13.2 %)
	+++	70 (37.0 %)	69 (11.1 %)
Initial laboratory results			
White blood cells, median (IQR), 10E9/L		12.7 (7.3)	10.8 (6.3)
C-reactive protein, median (IQR), mg/L		137.5 (130.5)	80 (140)
Treatment trajectories			
Length of stay, median (IQR)		3 (4)	2 (6)
90 days readmission, % (no.)		23.5 % (47)	21.0 % (161)
30 days intensive care, % (no.)		2.4 % (5)	2.4 % (18)
90 days mortality, % (no.)		9.5 % (19)	7.8 % (60)

Patients diagnosed with a UTI had a median length of stay of 3 days (IQR 4) and a 90-day mortality of 9.5 %.

### 3.2 Factors associated with UTIs

In the fully adjusted logistic regression analysis of patients’ reported new symptoms, we found that sensation of fever (OR 2.0), suprapubic or flank pain (OR 3.7), increased urinary frequency (OR 3.2), dysuria (OR 7.8), new urine retention (OR 6.4), new urine incontinence (OR 4.5), change of urine appearance (OR 3.9), and change of smell (OR 5.2) were all significantly associated with UTI (Table 2).

**Table 2:** Unadjusted, partially and fully adjusted logistic regression of significant factors associated with being diagnosed with a urinary tract infection when admitted to an emergency department with suspicion of infection.

**Patient characteristic**		**Unadjusted**			**Fully adjusted**		
n = 966		Odds ratio	(95 % conf.	interval)	Odds ratio	(95 % conf.	interval)
Age, years		1.015	1.01	1.03^a^	No confounders to adjust for		
Sex, male		1.23	0.899	1.686	Not significant in unadjusted analysis		
Comorbidities and Previous infections							
Previous urinary tract infection, n = 863		2.034	1.446	2.86^a^	1.692	1.146	2.5^a^
Chronic kidney disease		2.187	1.255	3.81^a^	1.706	0.957	3.041
Kidney stones or Previous kidney stones		2.545	1.278	5.07^a^	2.017	0.95	4.283^c^
Urine incontinence		2.319	1.045	5.146^a^	2.205	0.976	4.985
Urine retention or prostate hypertrophy		2.378	1.463	3.863^a^	1.512	0.858	2.664^c^
Indwelling urine catheter		5.726	3.619	9.061^a^	4.576	2.767	7.566^a^
Diabetes		1.617	1.113	2.351^a^	1.266	0.84	1.908
Pulmonary comorbidities		0.537	0.365	0.789^a^	0.499	0.338	0.736^a^
Previous lower respiratory tract infection, n = 863		0.602	0.429	0.846^a^	0.618	0.439	0.871^a^
Previous or current cancer of the urinary tract		3.042	1.85	5.002^a^	2.701	1.611	4.529^a^
Reported symptoms							
Sensation of fever		1.656	1.176	2.338^a^	2.044	1.423	2.937^a^
Suprapubic or flank pain		2.742	1.737	4.328^a^	3.678	2.234	6.053^a^
Increased urinary frequency, n = 922		3.081	2.153	4.408^a^	3.156	2.192	4.555^a^
Dysuria, n = 922		7.629	4.867	11.96^a^	7.801	4.919	12.37^a^
New urine retention, n = 922		6.169	3.759	10.13^a^	6.373	3.846	10.56^a^
New urine incontinence, n = 922		4.055	2.587	6.356^a^	4.457	2.808	7.073^a^
Change of urine appearance, n = 922		3.842	2.603	5.672^a^	3.885	2.554	5.91^a^
Change of urine smell, n = 922		4.606	2.877	7.373^a^	5.154	3.145	8.444^a^
Confusion, n = 927		1.571	1.097	2.249^a^	1.499	1.041	2.158^a^
Airway symptoms, n = 925		0.278	0.198	0.39^a^	0.301	0.212	0.426^a^
Cardiac symptoms, n = 924		0.3	0.187	0.48^a^	0.313	0.194	0.504^a^
Lost appetite, n = 926		1.429	1.025	1.993^a^	1.544	1.077	2.213^b^
Initial vital signs							
Respiratory rate, n = 960		0.956	0.921	0.99^a^	0.956	0.921	0.992^a^
Periferal oxygen saturation, %, n = 962		1.125	1.061	1.192^a^	1.143	1.07	1.221^a^
Mean arterial blood pressure, mmHg, n = 963		0.982	0.972	0.993^a^	0.984	0.973	0.994^a^
Temperature, °C, n = 961		1.527	1.307	1.784^a^	1.519	1.296	1.781^a^
Initial clinical examination							
Abnormal pulmonary stethoscopy, n = 911		0.348	0.237	0.511^a^	0.322	0.213	0.485^a^
Palpatory flank or suprapubic tenderness, n = 966		3.89	2.488	6.08^a^	4.321	2.647	7.053^a^
Initial urine dipstick results							
Urine white blood cells, n = 812	Negative	1(ref.)			1(ref.)		
	+	5.69	3.27	9.93^a^	5.99	3.37	10.64^a^
	++	11.92	7.19	19.74^a^	11.9	7.08	20.1^a^
	+++	14.91	7.91	28.11^a^	15.6	8.06	30.3^a^
	++++	25.94	13.36	50.38^a^	24.0	12.1	47.8^a^
Urine nitrite, n = 812	Negative						
	Positive	4.83	3.20	7.27^a^	4.66	3.03	7.16^a^
Urine blood, n = 811	Negative						
	+/− or +	3.80	2.22	6.51^a^	3.62	2.09	6.27^a^
	++	7.40	4.17	13.12^a^	6.59	3.67	11.8^a^
	+++	13.67	7.86	23.79^a^	12.0	6.80	21.3^a^
Urine protein, n = 812	Negative						
	+	1.60	1.00	2.57^a^	1.47	0.91	2.38
	++	3.40	2.20	5.25^a^	3.01	1.93	4.69^b^
	+++	8.75	4.84	15.83^a^	7.28	3.95	13.4^a^
Initial laboratory results							
Platelets, 10E9/L, n = 956		0.995	0.993	0.997^a^	0.995	0.993	0.997^a^
Lymphocytes, 10E9/L, n = 329		0.518	0.336	0.799^a^	0.534	0.339	0.84^a^
Hemoglobin, mmol/L		0.856	0.749	0.979^a^	0.909	0.785	1.052
White blood cells, 10E9/L	<3.5	1(ref.)			Not significant in unadjusted analysis		
	3.5–8.8	0.55	0.11	2.745			
	8.8–15.0	0.89	0.182	4.353			
	>15.0	1.535	0.311	7.584			
Creatinine, μmol/L	<60(m) 45(f)	1(ref.)			1(ref.)		
	60-105(m) 45–90(f)	2.008	0.781	5.167	2.094	0.812	5.403
	105(m) 90(f)	4.083	1.578	10.57^a^	3.519	1.341	9.235^a^
Bilirubin,μmol/L, n = 955	<5	1(ref.)			1(ref.)		
	5–25	3.53	1.754	7.106^a^	3.366	1.662	6.817^a^
	>25	3.588	1.267	10.17^a^	3.206	1.118	9,192^a^
C-reactive protein, mg/L	<8.8	1(ref.)			1(ref.)		
	8.8–50	1.873	0.812	4.319	1.787	0.758	4.217
	50–100	3.884	1.752	8.608^a^	3.412	1.503	7.743^b^
	101–200	4.718	2.192	10.16^a^	4.046	1.835	8.925^b^
	>200	5.481	2.501	12.01^a^	5.338	2.388	11.93^b^

a Significant variables.

b Significant variables that are non-significant after non-infected were excluded.

c Non-significant variables that are significant after non-infected were excluded.

Any indwelling urinary catheter was associated with an increased risk of having a UTI (OR 4.6), but nephrological comorbidities were not. Of the cancers, only urinary tract cancer was associated with UTIs (OR 2.7).

On clinical examination, suprapubic or flank pain (OR 4.3) was associated with UTI.

In our population, the UDA of WBC (1+ OR 6.0; 2+ OR 11.9; 3+ OR 15.6; 4+ OR 24.0), nitrite (OR 4.7), protein (1+ OR 1.5; 2+ OR 3.0; 3+ OR 7.3), and blood (1+ OR 3.6; 2+ OR 6.6; 3+ OR 12.0) were strongly associated with a diagnosis of UTI.

In the laboratory results, higher lymphocytes (OR 0.53) and platelets (OR 0.995) reduced the risk of a UTI. We could not find a significant association between WBC count and UTIs.

We initially found an association between UTI and C-reactive protein and UTI and loss of appetite, but when we excluded patients without infections, this association disappeared. Conversely, we found significant associations that were non-significant in the fully adjusted analysis when excluding the non-infected patients. This was the case for patients with a history of kidney stones (OR 2.6) and prior urine retention or prostate hypertrophy (OR 1.8).

### 3.3 Patient outcomes

The Length of stay, 90-day mortality, 90-day readmission, and 30-day intensive care unit admission were similar between the UTI and non-UTI patients, and we found no statistically significant difference. Furthermore, no difference in the likelihood of same-day discharge or length of stay was observed between patients with and without a UTI.

### 3.4 Subgroup analyses

In a sub-group analysis of UDA results, the strong association found in WBC, nitrite, protein, and blood was present regardless of sex. Notably, the association was generally stronger in men than in women. If patients without infection were excluded from the analysis, the association became even stronger (Table 3).

**Table 3:** Fully adjusted subgroup analysis of urinary dipistick association with urinary tract infections in males and females in the entire population and in the infected population.

		**Female**			**Male**		
		Odds ratio	(95 % conf.	interval)	Odds ratio	(95 % conf.	interval)
**Entire population**							
Urine white blood cells, n = 703	negative	1 (reference)					
	+	4.64	2.09	10.3	7.84	3.36	18.3
	++	6.07	2.78	13.3	23.0	10.9	48.5
	+++	10.6	4.18	26.8	23.8	8.95	63.3
	++++	18.4	6.15	55.1	30.4	12.0	76.8
Urine red blood cells, n = 702	negative	1 (reference)					
	+	3.96	1.84	8.51	3.33	1.46	7.58
	++	5.39	2.24	12.96	8.4	3.73	19.0
	+++	6.33	2.53	15.82	17.3	7.92	37.8
Urine nitrite, n = 703	negative	1 (reference)					
	positive	4.97	2.64	9.33	4.66	2.54	8.56
**Infected only**							
Urine white blood cells, n = 590	negative	1 (reference)					
	+	4.92	2.15	11.3	8.50	3.49	20.7
	++	5.80	2.58	13.1	28.8	12.94	64.0
	+++	11.9	4.35	32.3	19.3	7.20	51.9
	++++	30.3	8.28	111	37.2	13.40	103
Urine red blood cells, n = 590	negative	1 (reference)					
	+	3.30	1.51	7.20	3.49	1.51	8.08
	++	4.45	1.81	10.9	7.72	3.36	17.7
	+++	5.37	2.08	13.9	16.0	7.19	35.8
Urine nitrite, n = 590	negative	1 (reference)					
	positive	4.65	2.40	9.02	4.02	2.14	7.55

All p-values are below 0.05.

## 4 Discussion

### 4.1 Key results

In this pragmatic prospective observational study using expert panel assessment for diagnosis, one-fifth of acutely admitted patients with suspicion of infection to a medical ED were diagnosed with a UTI. Fever was the most common symptom (73 %), and increased urinary frequency, change in urine smell, and dysuria were present in about a third of the patients, respectively.

We found that clinical symptoms of a UTI, such as dysuria, new urine retention, change of urine smell and appearance, new urine incontinence, frequent urination, and suprapubic or flank pains, were strongly associated with being diagnosed with a UTI. The strongest associated factor was UDA for WBC, nitrite, and blood.

Our study had some limitations. Due to the exploratory study design, we did not take multiple testing into account. With 200 cases and 66 analyzed variables (34 are significantly associated), we have few events per variable, and some of our significantly associated variables could be due to multiple testing.

All patients were included during the day and evening, so a selection bias was introduced by not including at night and on weekends. Though mortality is known to be lower during weekdays in the daytime, we do not consider it to affect our results significantly [25].

Since it was required that the patient was able to give consent to the study, the prevalence of confusion is underreported in this cohort.

We generally had low levels of missing data; in most cases, we classified it as missing completely at random or missing at random. Besides blood lymphocytes, taken as a part of the standard laboratory panel at only one hospital and considered missing at random, the variable with the highest number of missing was UDA (16 %). Most were not taken due to time constraints, others due to patients being anuric at admission, which would be associated with more severe disease. Additionally, the missing data of anuric and oliguric patients in either group, who would likely have more blood and WBC in the urine due to inflammation, could have significantly reduced the association of our UDA results.

As no validated and generally accepted scoring systems or algorithms exist for UTIs, the parameters for the diagnosis were highly subjective for each expert. We moderated this by having the two experts assess every patient separately and agree on the diagnosis.

The slight male predominance we found in this cohort is atypical, unlike others who report a majority of women [26]. However, some large epidemiological surveys have shown a comparable male predominance in UTIs of 59.9 % [27].

In line with other studies, we established that classic UTI symptoms are strongly associated with a UTI diagnosis [28,29]. Dysuria is the symptom with the highest risk of a UTI diagnosis in our study, which is in line with other reports and is part of the UTISA (UTI Symptoms Assessment questionnaire) score for UTIs [10,21,28,30]. Urinary retention is a well-known risk factor for UTI, which our data confirm even in the case of the symptom being present for less than fourteen days [31]. Previous studies disagree on the association of increased urinary frequency and urinary incontinence with having a UTI, but our findings confirm that this association is present [28,30].

Reporting confusion was associated with UTI in this study, though it had a low odds ratio. Due to the requirement of being able to give consent, we have a probable selection bias that could have influenced this association. However, some studies have reported similar results [32,33].

Although these results should not surprise any clinician treating these patients, they indicate that even in a study where UTI symptoms are not obligatory for diagnosing a UTI, they are still highly associated with the diagnosis and should be considered when establishing diagnostic criteria. Additionally, our results underline the importance of getting a thorough medical history when trying to find the focus of a suspected infection.

The role of UDA in diagnosing UTI is disputed, generally due to low sensitivity and positive predictive value [18]. In our data, though, UDA for WBCs was the single factor with the highest odds ratio for being diagnosed with a UTI and remained so even when adjusting for confounders and sub-analyzing for sex.

We believe that the varying results of other studies reflect how UTI is defined and the population being studied. Due to our study design and UTI definition, we argue that our results indicate that UDA for WBC, blood, and nitrite can be an effective approach in identifying a focus in a patient suspected of having an infection. Our findings that the difference in the risk associated with a positive UDA WBC, nitrite, or blood varies with sex is in line with previous studies [18]. So, this difference persists even when confirmed bacteriuria was not requisite for a UTI diagnosis, indicating that earlier findings are not driven by the higher incidence of asymptomatic bacteriuria in females.

Furthermore, to our knowledge, no previous study has shown a strong association between UDA for blood and the diagnosis of UTI, which could add an additional level of diagnostic accuracy to UDA.

Our results show a high degree of generalizability in an ED setting due to the pragmatic study design with the inclusion of a broad selection of patients in a clinical setting, relatively few non-restrictive exclusion criteria, and a reasonably large sample size for a clinical trial. Moreover, our inclusion of three centers covering a diverse population evaluated by an expert panel of experienced infectious diseases and emergency medicine specialists makes these results highly generalizable nationally. Furthermore, though Denmark has a low ethnic diversity, our results are likely internationally generalizable to settings with populations of European descent, similar organization of their healthcare system, and disease burden.

## 5 Conclusion

Positive UDA for WBC, nitrite, and blood is a strongly associated factor for UTI in patients admitted to an ED with suspected infection. Clinical symptoms of a UTI are also a strong factor, and exploring the medical history of a patient suspected of having an infection is highly recommended.

This study is exploratory; as such, these results should be used for hypothesis generation and not for developing clinical guidelines. More research into these factors, especially UDA and similar point-of-care tests, is needed to increase diagnostic accuracy in the ED. Preferably with a similarly pragmatic, unbiased, and clinical-based definition of a UTI to better represent the clinical reality.

## Funding source

This study was funded by the 10.13039/501100006356University of Southern Denmark (no grant number), the joint research pool of the Region of Southern Denmark and the Region of Zealand under Grant A797, 10.13039/501100004196Odense University Hospital (no grant number), Hospital Sønderjylland (no grant number), and Gundhild Andersen's Foundation under Grant A5733.

The sponsors did not influence study design, research goals, data collection, data analysis, data interpretation, or any part of this article.

## Ethics and consent

The INDEED study was approved by the Regional Committees on Health Research Ethics for Southern Denmark (ID S-20200188). The study is also reported to the Danish Data Protection Agency (no. 20/60508), registered in ClinicalTrial.com (NCT04667195), and is part of Patient Data Explorative Networking (OPEN). The INDEED study was done in complete concordance with the Declaration of Helsinki. Every participant provided written consent for participation and publication of results.

## Data availability statement

Data is unavailable as Danish Data Protection laws prohibit dissemination of patient data.

## CRediT authorship contribution statement

**Mathias Amdi Hertz:** Writing – review & editing, Writing – original draft, Investigation, Funding acquisition, Formal analysis, Data curation, Conceptualization. **Helene Skjøt-Arkil:** Writing – review & editing, Supervision, Resources, Project administration, Funding acquisition, Data curation, Conceptualization. **Anne Heltborg:** Writing – review & editing, Data curation, Conceptualization. **Morten Hjarnø Lorentzen:** Writing – review & editing, Data curation, Conceptualization. **Mariana Bichuette Cartuliares:** Writing – review & editing, Data curation, Conceptualization. **Flemming S. Rosenvinge:** Writing – review & editing, Conceptualization. **Stig Lønberg Nielsen:** Writing – review & editing, Supervision. **Christian Backer Mogensen:** Writing – review & editing, Supervision, Project administration, Funding acquisition, Conceptualization. **Isik Somuncu Johansen:** Writing – review & editing, Supervision.

## Declaration of competing interest

The authors declare that they have no known competing financial interests or personal relationships that could have appeared to influence the work reported in this paper.

## List of abbreviations

UTI -: urinary tract infection
UDA –: urine dipstick analysis
WBC –: white blood cells
