# Supplementary material for: Clinical characteristics, factors associated with urinary tract infection and outcome in acutely admitted patients with infection; an exploratory cross-sectional cohort study
Source: Heliyon. 2024 Jun 11;10(12):e32815. doi: 10.1016/j.heliyon.2024.e32815 (PMC11231541; doi:10.1016/j.heliyon.2024.e32815)
Supplement: Multimedia component 1 [file mmc1.docx]

| Appendix 1. Analysed variables not included in table 2 |  |  |  |  |  |  |  |
| --- | --- | --- | --- | --- | --- | --- | --- |
| **Patient Characteristics** |  | **Cause for exclusion from table 2** |  | **Unadjusted logstic regression** | | | |
|  |  |  |  | Odds ratio | (95% conf. | interval) | |
| Comorbidities and Previous infections |  |  |  |  |  |  |  |
| Previous cellulitis, n=856 |  | Non-significant in unadjusted analysis |  | 0.624 | 0.367 | 1.060 |  |
| Neurological Comorbidities n=966 |  | Non-significant in unadjusted analysis |  | 0.830 | 0.546 | 1.262 |  |
| Osteoporosis |  | Non-significant in unadjusted analysis |  | 1.041 | 0.638 | 1.698 |  |
| Non diabetic or osteoporosis endocrinological comorbidities |  | Non-significant in unadjusted analysis |  | 1.055 | 0.572 | 1.948 |  |
| Non kidney stone or chronic kidney disease nephrological comorbidities |  | Non-significant in unadjusted analysis |  | 1.801 | 0.958 | 3.386 |  |
| Cardiological comorbidities |  | Non-significant in unadjusted analysis |  | 0.896 | 0.652 | 1.232 |  |
| Previous or current non urinary tract, abdominal, or gynecological cancer |  | Non-significant in unadjusted analysis |  | 1.395 | 0.902 | 2.159 |  |
| Previous or current abdominal og gynecological cancer |  | Non-significant in unadjusted analysis |  | 1.454 | 0.815 | 2.596 |  |
| Gastrointestinal comorbidities |  | Non-significant in unadjusted analysis |  | 0.953 | 0.568 | 1.597 |  |
| Rheumatological comorbidities |  | Non-significant in unadjusted analysis |  | 0.757 | 0.456 | 1.259 |  |
|  |  |  |  |  |  |  |  |
| Reported symptoms |  |  |  |  |  |  |  |
| Nausea |  | Non-significant in unadjusted analysis |  | 1.181 | 0.844 | 1.651 |  |
| Vomiting |  | Non-significant in unadjusted analysis |  | 1.244 | 0.851 | 1.820 |  |
| Diarrhea |  | Non-significant in unadjusted analysis |  | 0.686 | 0.418 | 1.127 |  |
| Obstipation |  | Non-significant in unadjusted analysis |  | 1.378 | 0.763 | 2.489 |  |
| Joint and muscle pain |  | Non-significant in unadjusted analysis |  | 0.846 | 0.594 | 1.205 |  |
| Backpain |  | Non-significant in unadjusted analysis |  | 1.220 | 0.787 | 1.890 |  |
|  |  |  |  |  |  |  |  |
| Initial urine dipstick results |  |  |  |  |  |  |  |
| Urine glucose | + | Non-significant in unadjusted analysis |  | 1.294 | 0.574 | 2.914 |  |
|  | ++ | Non-significant in unadjusted analysis |  | 1 |  |  |  |
|  | +++ | Non-significant in unadjusted analysis |  | 0.803 | 0.292 | 2.211 |  |
|  | ++++ | Non-significant in unadjusted analysis |  | 1 |  |  |  |
| Urine ketones | + | Non-significant in unadjusted analysis |  | 1.038 | 0.625 | 1.725 |  |
|  | ++ | Non-significant in unadjusted analysis |  | 1.175 | 0.624 | 2.212 |  |
|  | +++ | Non-significant in unadjusted analysis |  | 0.676 | 0.290 | 1.577 |  |
|  | ++++ | Non-significant in unadjusted analysis |  | 1.811 | 0.582 | 5.637 |  |
|  | >++++ | Non-significant in unadjusted analysis |  | 1.449 | 0.429 | 4.893 |  |
|  |  |  |  |  |  |  |  |
| Initial laboratory results |  |  |  |  |  |  |  |
| Hematocrite |  | Correlates with hemoglobin |  | 0.005 | 0.000 | 0.158 | a |
| Neutrophilocytes |  | Correlates with white blood cells |  | 1.072 | 1.043 | 1.102 | a |
| Blood urea nitrogen |  | Correlates with creatinine |  | 1.046 | 1.021 | 1.073 | a |
| Sodium |  | Non-significant without extreme outliers |  | 0.955 | 0.921 | 0.990 | a |
| Albumine | <34 | Non-significant in unadjusted analysis |  | 1(ref.) |  |  |  |
|  | 34-50 |  |  | 0.746 | 0.471 | 1.183 |  |
|  | >50 |  |  | 1 |  |  |  |
| Glucose | <3.1 | Non-significant in unadjusted analysis |  | 1 |  |  |  |
|  | 3.1-11 |  |  | 1(ref.) |  |  |  |
|  | 11.1-16 |  |  | 1.084 | 0.544 | 2.157 |  |
|  |  |  |  |  |  |  |  |
| ”a” denotes significant variables. |  |  |  |  |  |  |  |
